# Early microvascular dysfunction in cerebral small vessel disease is not detectable on 3.0 Tesla magnetic resonance imaging: a longitudinal study in spontaneously hypertensive stroke-prone rats

**DOI:** 10.1186/2040-7378-5-8

**Published:** 2013-06-25

**Authors:** Stine Mencl, Cornelia Garz, Solveig Niklass, Holger Braun, Eva Göb, György Homola, Hans-Jochen Heinze, Klaus G Reymann, Christoph Kleinschnitz, Stefanie Schreiber

**Affiliations:** 1 Department of Neurology, University Hospital of Würzburg, Würzburg, Germany; 2 Department of Neurology, Otto-von-Guericke-University, Magdeburg, Germany; 3 Leibniz Institute for Neurobiology, Magdeburg, Germany; 4 German Center for Neurodegenerative Diseases (DZNE), Magdeburg, Germany; 5 Department of Neuroradiology, University Hospital of Würzburg, Würzburg, Germany

**Keywords:** Cerebral small vessel disease, SHRSP, MRI

## Abstract

**Background:**

Human cerebral small vessel disease (CSVD) has distinct histopathologic and imaging findings in its advanced stages. In spontaneously hypertensive stroke-prone rats (SHRSP), a well-established animal model of CSVD, we recently demonstrated that cerebral microangiopathy is initiated by early microvascular dysfunction leading to the breakdown of the blood–brain barrier and an activated coagulatory state resulting in capillary and arteriolar erythrocyte accumulations (stases). In the present study, we investigated whether initial microvascular dysfunction and other stages of the pathologic CSVD cascade can be detected by serial magnetic resonance imaging (MRI).

**Findings:**

Fourteen SHRSP and three control (Wistar) rats (aged 26–44 weeks) were investigated biweekly by 3.0 Tesla (3 T) MRI. After perfusion, brains were stained with hematoxylin–eosin and histology was correlated with MRI data. Three SHRSP developed terminal CSVD stages including cortical, hippocampal, and striatal infarcts and macrohemorrhages, which could be detected consistently by MRI. Corresponding histology showed small vessel thromboses and increased numbers of small perivascular bleeds in the infarcted areas. However, 3 T MRI failed to visualize intravascular erythrocyte accumulations, even in those brain regions with the highest densities of affected vessels and the largest vessels affected by stases, as well as failing to detect small perivascular bleeds.

**Conclusion:**

Serial MRI at a field strength of 3 T failed to detect the initial microvascular dysfunction and subsequent small perivascular bleeds in SHRSP; only terminal stages of cerebral microangiopathy were reliably detected. Further investigations at higher magnetic field strengths (7 T) using blood- and flow-sensitive sequences are currently underway.

## Introduction

Human cerebral small vessel disease (CSVD), a major cause of stroke-like symptoms and dementia, is associated with lacunar infarcts, lacunes, microbleeds, enlarged perivascular spaces, white matter lesions of the cerebral and cerebellar hemispheres, and primary intracerebral hemorrhages [1,2]. These are associated with distinct histopathologic and imaging findings in advanced and terminal stages of the disease. To what extent the underlying histopathology (including degenerative small vessel wall changes and the presence of trabeculated cavities surrounded by gliosis with small vessel occlusions and wall enlargements [3,4]), corresponds to imaging findings remains unclear.

We have recently conducted several longitudinal studies using spontaneously hypertensive stroke-prone rats (SHRSP), an animal model that is widely accepted to be the most valid model of human CSVD [1,5,6], and found that CSVD is initiated by subtle, early microvascular dysfunction that ultimately leads to a breakdown in the blood–brain barrier, an activated coagulatory state, and an intraluminal accumulation of erythrocytes that are visible as “stases” using conventional histology [7-11]. The resultant increasing vessel wall fragility leads to small perivascular bleeds and reactive (complete) hyaline fibrin thromboses with associated infarcts [7].

The aim of our current study was to investigate whether stases (or associated phenomena), which are indicative of early microvascular dysfunction in SHRSP, are detectable using 3.0 Tesla (3 T) MRI. This imaging technique has been used previously in rat studies [12] and is employed as a standard diagnostic in the clinical setting.

## Materials and methods

All animal procedures were approved by the local Ethical Committees in accordance with the animal protection guidelines and European Communities Council guidelines (55.2-2531.01-74/11; 42502-2-1148 DZNE). Fourteen male SHRSP and 3 control Wistar rats (Charles River Laboratories International Inc., Wilmington, MA, USA) underwent biweekly 3 T MRI (Siemens MAGNETOM Trio, Erlangen, Germany) from the age of 26 weeks. Animals were anesthetized (2% isoflurane), and respiratory frequency and body temperature were monitored. Imaging was performed using a 4-channel array rat head coil (RAPID Biomedical, Rimpar, Germany) with the following sequences/protocols: T1-weighted (w) (repetition time (TR) 700 ms, echo time (TE) 13 ms, slice thickness (SLT) 0.9 mm, in plane resolution 0.2 × 0.2 mm), T2w (TR 4900 ms, TE 110 ms, SLT 1.0 mm, in plane resolution 0.1 × 0.1 mm), T2w constructive interference in steady-state three-dimension (CISS 3D; TR 8.8 ms, TE 3.8 ms, SLT 0.3 mm, in plane resolution 0.3 × 0.3 mm), fluid-attenuated inversion recovery (FLAIR; TR 7500 ms, TE 90 ms, inversion time 2100 ms, flip angle (FA) 150°, SLT 2.0 mm, in plane resolution 0.2 × 0.2 mm), gradient echo (GRE; TR 21 ms, TE 6.1 ms, FA 25°, SLT 1.0 mm, in plane resolution 0.2 × 0.2 mm). MRIs were assessed qualitatively by an experienced neuroradiologist (G.H.).

The rats were transcardially perfused when they showed evidence of anxiety, aggression, decreased spontaneous activity, moving deficits, coordination failure, falling to one side, or weight loss (>10% weekly), or at an age of 44 weeks. Perfusion was performed with 120 ml phosphate-buffered saline, followed by 120 ml of 4% paraformaldehyde (PFA); brains were removed, fixed in 4% PFA for 48 h at 4°C and placed for cryoprotection for 6 days into 30% sucrose before being frozen in methylbutane at −80°C. Coronal slices (30 μm, 30–33 slices per animal) of the whole brains of all animals who underwent MRI and six additional Wistar control rats (aged 44 weeks) were stained with hematoxylin–eosin, and the density of vessels affected by stases (given as mean out of 50 fields of view (FoV) per brain region, magnification × 200) and the occurrence and extent of perivascular small bleeds, thromboses/associated infarcts, and macrohemorrhages were assessed in all brain regions including the basal ganglia, thalamus, cortical regions, hippocampus, and corpus callosum. Additionally, the luminal diameter of the small vessels affected by stases and the diameter of all bleeds were measured.

## Results

**Figure 1:**
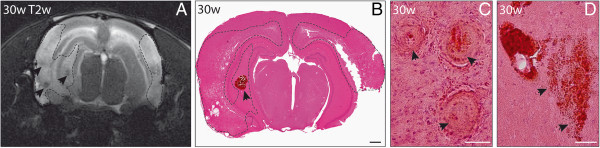
**Cortical and hippocampal small vessel thromboses, small perivascular bleeds, infarcts, and macrohemorrhages in spontaneously hypertensive stroke-prone rats (SHRSP).** T2-weighted (T2w) sequences (**A**, distance from bregma −5 mm) and hematoxylin–eosin staining (**B**) with coronal slice orientation performed at the age of 30 weeks in SHRSP K1R1 showed bilateral cortical and hippocampal infarcts (dashed outline in **A**, **B**) as well as macrohemorrhages (black arrowheads in **A**, **B**). The corresponding histology displayed small vessel thromboses (black arrowheads in **C**) and a high number of small perivascular bleeds within the infarcted areas (exemplary ones marked with black arrowheads in **D**). w – age in weeks; Scale bar in **B**: 1000 μm; Scale bar in **C** and **D**: 50 μm.

**Figure 2:**
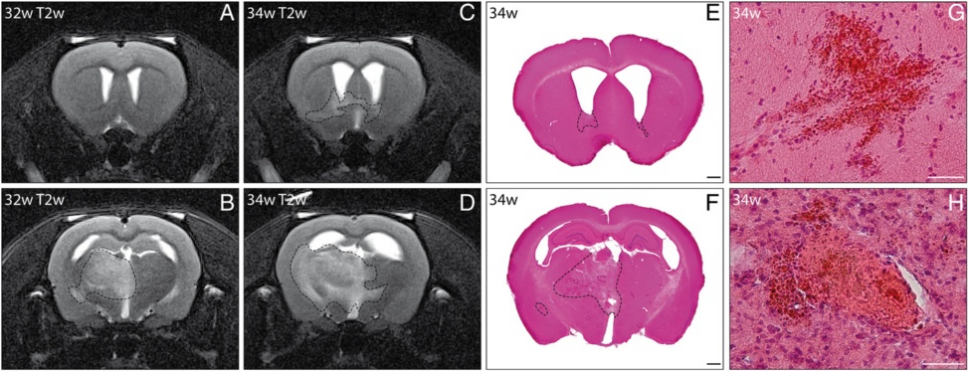
**Serial brain magnetic resonance imaging (MRI) revealed infarct progression within 2 weeks in spontaneously hypertensive stroke-prone rats (SHRSP).** T2-weighted (T2w) sequences (**A–D**) with coronal slice orientation were performed at the age of 32 weeks (**A**, **B**, DfB 0.7 mm, −2.3 mm) and 34 weeks (**C**, **D**, DfB 0.5 mm, −2.6 mm) in SHRSP K2R2 and revealed an infarct progression in the basal ganglia with midline shift (**D**) over time. Both MRI scans and the corresponding histology (**E, F**) showed a nearly panhemispheric infarct (dashed outline in **B–F**). Histology revealed a high number of small perivascular hemorrhages (**G**) and small vessel thromboses in the infarcted area (**H**). w – age in weeks; DfB – distance from bregma; Scale bar in **E** and **F**: 1000 μm. Scale bar in **G** and **H**: 50 μm.

**Figure 3:**
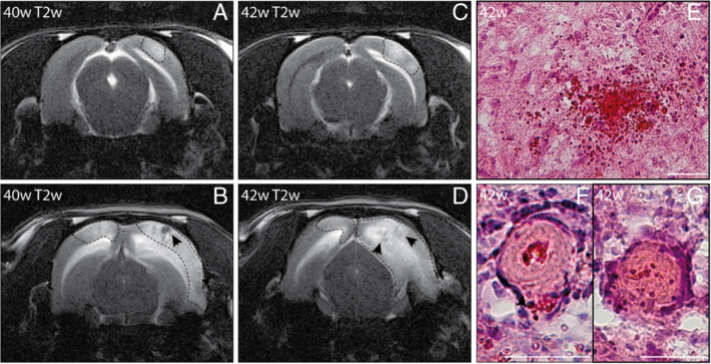
**Infarct progression and macrohemorrhages in spontaneously hypertensive stroke-prone rats (SHRSP).** T2-weighted (T2w) sequences (**A–D**) with coronal slice orientation were performed at the age of 40 weeks (**A**, **B**, DfB −7.3 mm, −5.6 mm) and 42 weeks (**C**, **D**, DfB −6.5 mm, −6.1 mm) in SHRSP K1R2 revealed a progression of bilateral cortical infarcts (dashed outline in **A–D**) and the embedded macrohemorrhage (black arrowheads in **B**, **D**) within 2 weeks. Histology showed a high number of small perivascular bleeds (**E**) and small thromboses in the infarcted area **(F**). w – age in weeks; DfB – distance from bregma. Scale bar in **E–G**: 50 μm.

**Figure 4:**
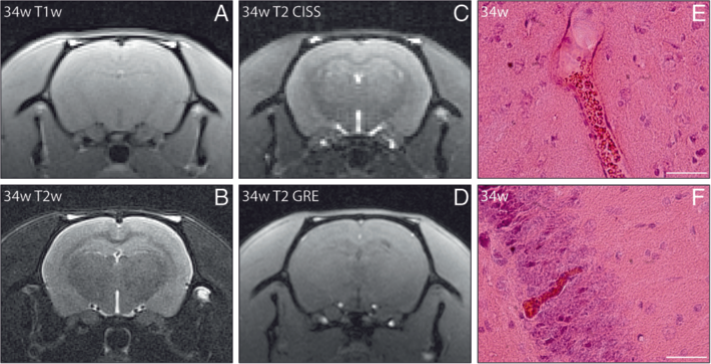
**No pathology on magnetic resonance imaging in spontaneously hypertensive stroke-prone rats (SHRSP) with only stases.** T1-weighted (T1w; **A**), T2-weighted (T2w; **B**), T2 constructive interference in steady state (**C**) and T2 gradient echo (**D**) (**A–D**: DfB −3.2 mm) sequences with coronal slice orientation performed at the age of 34 weeks in SHRSP K2R3 showed no abnormal findings. Histology at the age of 34 weeks displayed arteriolar stases in the cortex (**E**) and capillary stases in the hippocampus (**F**). w – age in weeks; DfB – distance from bregma. Scale bar in **E** and **F**: 50 μm.

Three of the 14 SHRSP investigated exhibited T2w hyperintensities, predominantly localized in the parietal cortex, basal ganglia, and hippocampus, indicating infarcts first detectable on MRI at the ages of 30, 32, and 40 weeks (Figures 1, 2 and 3) and extending over approximately 2 weeks (Figures 2 and 3). In two of these SHRSP, cortical (2200 μm diameter (Figure 1) and 1100 μm diameter (Figure 3)) and hippocampal (1600 μm diameter (Figure 1)) macrohemorrhages were embedded between the T2w hyperintensities. The MRI data were confirmed by histology, demonstrating small vessel thromboses with evidence of stases in the infarcted regions of the three SHRSP (Figures 1, 2 and 3). Compared with the SHRSP without infarcts/macrohemorrhages (1–25 small perivascular bleeds per animal), up to a 20-fold higher number of small perivascular bleeds (75–510 per animal) were detected in the infarcted regions (Figures 1, 2 and 3). However, only two of these three SHRSP showed weight loss and behavior changes, while one (Figure 3) was free from any of these symptoms. One SHRSP developed behavior abnormalities and weight loss without any detectable MRI pathology; histology revealed only a few stases, predominantly in the cortical (0.56 per FoV) and hippocampal (0.40 per FoV) capillary bed and in the cortical, and striatal arterioles (0.44 per FoV; Figure 4).

The remaining SHRSP (n = 10) and all investigated Wistar control rats showed no MRI pathologies, no behavior abnormalities/weight loss and no thromboses/infarcts or macrohemorrhages. The histology of these rats revealed capillary (mean: 4.5 per FoV per animal) and, to a far lesser extent, arteriolar stases (mean: 0.08 per FoV per animal) in all investigated brain regions; however, even in animals with the highest density of vessels with capillary (22.18 per FoV per animal) or arteriolar (0.36 per FoV per animal) stases, no MRI abnormalities were detectable and the diameter of the affected small vessels did not exceed 60 μm. In nine of these 10 SHRSP (90%) and four of the nine controls (44%), small perivascular bleeds with a maximal diameter of 360 μm (SHRSP) or 125 μm (Wistar rats), respectively, were verifiable in all investigated brain regions; most of these small bleeds did not exceed a diameter of 50 μm.

In conclusion, 3 T MRI effectively detected infarcts and macrohemorrhages exceeding 800 μm in diameter in SHRSP, but failed to detect small perivascular bleeds and arterial stases in SHRSP and controls. Behavior changes and weight loss were unreliable predictors of small perivascular bleeds and stases in this study; a finding that requires further evaluation in a larger cohort of animals. The burden and increasing diameter of small perivascular bleeds in infarcted regions support our concept of a pathologic cascade of CSVD in SHRSP [13]. Further investigations at higher magnetic field strengths (7 T) using blood- and flow-sensitive sequences are currently underway to increase the diagnostic sensitivity of this MRI technique for early microvascular dysfunction.

## Abbreviations

CSVD: Cerebral small vessel disease; CISS 3D: Constructive interference in steady-state three-dimension; DfB: Distance from bregma; FA: Flip angle; FLAIR: Fluid-attenuated inversion recovery; FoV: Field of view; GRE: Gradient echo; MRI: Magnetic resonance imaging; PFA: Paraformaldehyde; SHRSP: Spontaneously hypertensive stroke-prone rats; SLT: Slice thickness; T: Tesla; T1w: T1-weighted; T2w: T2-weighted; TE: Echo time; TR: Repetition time.

## Competing interests

The authors declare that they have no competing interests.

## Authors’ contributions

SS directed the study, designed experiments, analyzed data and drafted the manuscript; SM designed and performed experiments, analyzed data, and drafted the manuscript; CG and HB designed and performed experiments, analyzed data, and made major contributions to the manuscript writing; EG designed and performed experiments; SN performed experiments; GH provided specific input to MRI experiments including experimental design and contributed to the manuscript writing; CK, HJH, and KGR funded the study, designed experiments and contributed to manuscript writing. All authors read and approved the final manuscript.
